# Straw mulching for enhanced water use efficiency and economic returns from soybean fields in the Loess Plateau China

**DOI:** 10.1038/s41598-022-21141-3

**Published:** 2022-10-12

**Authors:** Feng Li, Guohong Zhang, Juan Chen, Yali Song, Zhiguang Geng, Kefu Li, Kadambot H. M. Siddique

**Affiliations:** 1 Qingyang Academy of Agricultural Sciences, Qingyang, Gansu 745000 People’s Republic of China; 2 Institute of Dryland Agriculture, Gansu Academy of Agricultural Sciences, Lanzhou, Gansu 730070 People’s Republic of China; 3 Institute of Economic Crops and Beer Materials, Gansu Academy of Agricultural Sciences, Lanzhou, Gansu 730070 People’s Republic of China; 4 The UWA Institute of Agriculture and School of Agriculture & Environment, The University of Western Australia, M082, LB 5005, Perth, WA 6001 Australia

**Keywords:** Agroecology, Environmental sciences

## Abstract

Water shortages threaten agricultural sustainability in the semi-arid areas of the Loess Plateau. Judicious mulching management can improve water conservation practices to alleviate this issue while increasing crop productivity. We investigated the effect of straw strip mulching and film mulching on soil water consumption, temperature, growth, grain yield, and economic income of soybean [*Glycine max(Linn.)* Merr.] from 2017 to 2018 in Qingyang on the semi-arid Loess Plateau in China using four treatments: (a) alternating ridges and furrows with ridges mulched with white polyethylene film (PMP), (b) alternating flat and bare land with only the plat mulched by white polyethylene film (PMF), (c) alternating strips mulched with maize (*Zea mays* L.) straw (SM), and (d) traditional land planting without mulching (CK). The mulching treatments (PMP, PMF, and SM) increased soil water consumption and soil water use efficiency. The SM, PMF, and PMP treatments had 12.3–12.5, 16.8–22.1, and 23.2–24.2 mm higher soil water consumption (0–120 cm depth) than CK, most of which occurred in the 60–120 cm soil layer. Compared with CK, PMP and PMF significantly increased soil temperature by 1.30–1.31 °C and 0.76–1.00 °C, soybean grain yield by 38.6–39.0 % and 38.8–44.2 %, and water use efficiency (WUE) by 27.7–32.8 % and 30.8–37.5 %, respectively, while SM significantly decreased soil temperature by 0.96–1.15 °C, and increased grain yield by 21.8–25.4 % and WUE by 16.9–21.9 %. PMP and PMF did not significantly change soil water consumption, WUE, or grain yield. The SM treatment increased net income by 501.3–691.7 and 1914.5–2244.9 CNY ha^−1^ relative to PMP and CK, respectively, but PMF and SM did not significantly differ. Therefore, the SM system could help increase grain yields and economic returns in dryland soybean production, avoiding the adverse effects of the increasingly popular plastic mulching approach.

## Introduction

The Loess Plateau is located in the upper and middle reaches of the Yellow River in northern China, covering ~ 640,000 km^2^, with an average elevation of ~ 1200 m^1^. It has a typical continental monsoon climate, where winters are cold and dry, and most rainfall occurs during summer (June to September); annual precipitation is approximately 400 mm usually with heavy rain^2^. During summer, 50 % of soil moisture is lost through transpiration and evaporation due to intense radiation^2^. As a result, early crop growth experiences low temperatures and water shortages, limiting agricultural production^3^.

Plastic film is used widely in the semi-arid areas of the Loess Plateau to increase soil temperatures and retain soil moisture during crop production. Plastic film mulch increases the accumulation of soil thermal time during the seedling stage and improves water use efficiency (WUE) by promoting root growth and root activity^4–6^, enabling early sowing and early season growth^7,8^. Liu et al. revealed that mulching (especially plastic film) increases water use efficiency (WUE) and grain yield and decreases N leaching losses in dry farmland^9^. In a three-year study, Li et al. reported that mulching significantly increased maize yields from 13.0 to 15.0 % and WUE from 9.8 to 11.6 %^10^. Anzalone et al. and Summers et al. found that plastic film mulching reduced weed pressure and insect pests^11,12^. In contrast, residual plastic film is harmful to general soil water movement, limiting soil water absorption by crops and polluting farmland with mulch^10,13^. Global polyethylene consumption increased from 4.4 million tons in 2012 to 7.4 million tons in 2019, but is poorly recycled, resulting in the accumulation of plastic debris in soil^14^. The phthalates in plastic film area potential environmental and human health threat^15^. Zou et al. investigated the effect of residual film mulch levels on tomato growth and fruit quality, reporting a sharp decline in growth indexes, dry biomass, and yield with > 80 kg ha^−1^ of residual film mulch, and fruit shape index, organic acid, and lycopene decreased with increasing residual film mulch^16^. Hu et al. reported that plastic film residues of about 600 kg ha^−1^ decreased the root to shoot ratio during corn tasseling^17^.

The two main methods of straw returning for sustaining crop productivity and soil fertility in China involve incorporating straw into the top soil or mulching straw on the soil surface^18^. Straw mulch application can increase crop yield and economic benefit in wheat, millet, sorghum, and maize by decreasing evapotranspiration and soil water consumption and increasing WUE in dryland areas, but the yield results vary for wheat, maize, and potato^19–26^. In addition, straw mulch can improve soil status by enhancing microbial biomass, microbial activity, and potential N availability (and mitigate environmental pollution by controlling heavy metal contamination through surface runoff and reduced CH_4_ emissions^27–29^. Xue et al. showed that straw mulch enhanced soybean yield, yield components, photosynthetic pigments, and enzymatic activities to mitigate drought-induced oxidative damage^30^. Thus, straw mulch application might be an effective strategy for soybean cultivation in semi-arid areas on the Loess Plateau.

Soybean is commonly used in rotations in semi-arid areas of the Loess Plateau. However, no studies have investigated technologies other than plastic film mulch for improving soybean production and sustainability in this region. Here, we investigated the effect of various mulching practices on soybean production over two consecutive years, in the present research, we focus on determining: (1) investigate the effects of film mulching and strip mulching on soil temperature, soil water consumption and water use efficiency; (2) assess the effects of film mulching and strip mulching on grain yield, yield components of soybean and economic returns.

## Materials and methods

### Site description

The experiment was under taken from 2017 to 2018 in soybean fieldsin Hesheng town, Qingyang city of Gansu Province in China (35°25′ N, 107°47′ E; altitude: 1233.4 m). The climate is considered arid to semi-arid with annual average air temperature, annual sunshine hours, and an average frost-free period of 9.0 °C, 2369.1 h, and 168.2 d, respectively. The average annual precipitation is 568.9 mm, with nearly 60 % occurring between July to September; thus, the site is prone to drought in spring. The soil is aloessial soil, with an average bulk density of 1.25 g cm^−3^ (0–30 cm). The soil physicochemical properties (0–20 cm) are 14.43 g kg^−1^ soil organic matter, 0.99 g kg^−1^ total N, 11.42 mg kg^−1^ available P, and 153.83 mg kg^−1^ available K.

### Precipitation

Rainfall during the soybean growing season was 395.3 mm in 2017 and 413.0 mm in 2018. From 2011 to 2018, the annual average precipitation was 371.5 mm (Table 1). In 2017 and 2018, rainfall from sowing to flowering was 55.4 mm and 15.7 mm higher than average, respectively, providing adequate soil water for seedling growth.

**Table 1:** Precipitation during the 2017 and 2018 soybean growingseasons in Hesheng Town of Gansu Province, China.

Period	Precipitation (mm)		
	2017	2018	Mean 2011–2018
Sowing to flowering	144.7	105.7	89.3
Flowering to podset	61.3	136.4	82.1
Podset to podfill	48.2	81.9	63.9
Podfill to maturity	141.1	89.0	136.2
Total	395.3	413.0	371.5

In 2017, the rainfall from flowering to podset was lower than average, which affected flower formation and podset.

### Experimental design

**Figure 1:**
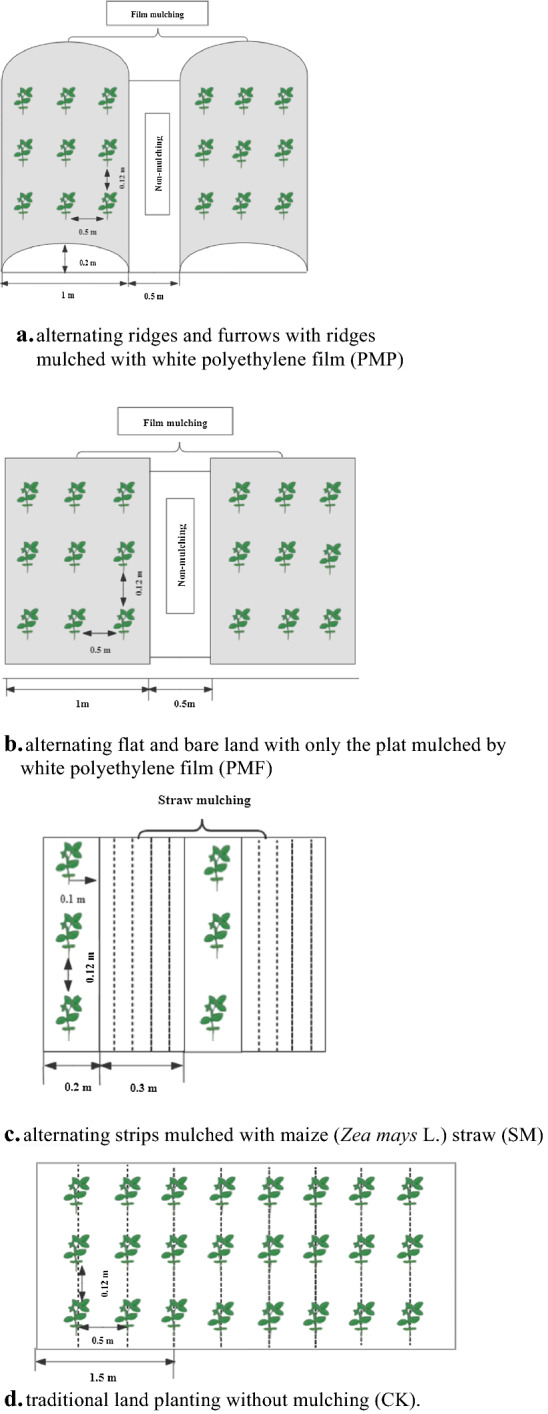
Schematic diagram of different cultivation practices. (**a**) alternating ridges and furrows with ridges mulched with white polyethylene film (PMP). (**b**) Alternating flat and bare land with only the plat mulched by white polyethylene film (PMF). (**c**) Alternating strips mulched with maize (*Zea mays* L.) straw (SM). (**d**) Traditional land planting without mulching (CK).

This study had four treatments: (a) alternating large ridges (1.0 m width, 0.2 m height) and small furrows (0.5 m width, 0.1 m depth) with the ridges mulched with white polyethylene film (PMP)—three rows of soybean were planted on the ridges, with 0.5 m between rows (Fig. 1a); (b) alternating mulched row (1.0 m width) with white polyethylene film and bare (0.5 m width) without ridges (PMF)—three rows of soybean were planted in the mulched strips with 0.5 m between rows (Fig. 1b); (c) alternating strips (0.3 m width) mulched with maize straw and bare plots (0.2 m width) with no ridges (SM)—soybean was planted in non-mulched strips with 0.5 m between rows (Fig. 1c); (d) traditional bare land planting (no mulch) with 0.5 m between rows (CK) (Fig. 1d). The experiment had a completely randomized block design with three replicates, with each plot 15 m long by 7 m wide. Each treatment received basal fertilization with 120.0 kg P_2_O_5_ ha^−1^ as ordinary superphosphate and 75 kg N ha^−1^ as urea. The soybean cultivar Longhuang No. 3 was planted at 165000 plants ha^−1^ with a 12 cm inter-plant spacing on 28 April 2017 (first growing season) and 6 May 2018 (second growing season). The previous crop was winter wheat, which yielded 6280.5 kg ha^−1^ in 2017 and 6542.1 kg ha^−1^ in 2018. For the PMP treatment, 100 cm wide ridges and 50 cm wide furrows were created one week before planting, with the ridges covered with transparent plastic film (polyethylene; 0.008 mm thickness and 140 cm width) before sowing soybean in shallow holes. For the PMF treatment, the alternating mulched rows (1.0 m width) with white polyethylene film and bare soil (0.5 m width) without ridges used 120 cm wide plastic film. For the SM treatment, maize straw (7500 kg ha^−1^) was applied immediately after seeding as mulch. Soybean for all treatments was sown manually in shallow holes 12 cm apart within rows, and the sedlings were thinned at the growth stage of V1 (1 node stage). Harvest occurred in late September. The crop received no supplemental irrigation during the experimental seasons. Weeds were controlled by hand; no obvious pests or diseases were observed.

### Sampling and measurements

#### Soil temperature and moisture

A set of mercury-in-glass geothermometers with bent stems (HY-1 Thermal Instruments, Kunshan, China) were placed between two plants within a row at 5, 10, 15, 20, and 25 cm depth to measure soil temperature. During each growing season, soil temperature was recorded at 700, 1400, and 1900 h on typical sunny days (seedling (V2), flowering (R1), pod-setting (R4), podfilling (R6), maturity (R8)) (Fehrand Caviness, 1977). The average value of three temperatures in the morning, midday, and evening was taken as the daily temperature. Soil samples were collected in 20 cm increments to a depth of 120 cm prior to sowing and at maturity using an auger at three locations within each plot. The samples were homogenized and brought to the laboratory for analysis. To determine soil water content (SW; Eq. 1), the samples were weighed before and after oven drying (105 °C, 24 h). Similarly, to determine soil bulk density ρ (g cm^-3^) using the ring knife method (gravimetrically) (Eq. 2), soil samples were collected in 20 cm increments to a depth of 120 cm prior to sowing and at maturity at three locations within each plot by taking 250 cm^3^ (8 cm diameter, 5 cm height) cylindrical cores, which were oven-dried (105 °C, dry to constant weight).

Soil water content was calculated as^31^:

1 $$SW = \sum\nolimits_{{\text{i}}}^{{\text{n}}} {{\text{h}}_{{\text{i}}} } * \rho_{{\text{i}}} * \omega_{{\text{i}}}$$

where *SW* (mm) is soil water storage, *h*_*i*_ is soil depth (mm), *ρ*_*i*_ is soil bulk density (g cm^−3^), *ω*_*i*_ is percentage soil moisture by weight (%), *n* is the number of soil layers, and *i* = 20, 40, 60… 120.

Soil bulk density was calculated as follows^32^:

2 $${\rm P}\mathrm{i}=\frac{\mathrm{mi}}{\mathrm{Vi}}$$

where *mi* is the mass of dry soil (g), *Vi* is the bulk soil volume (250 cm^3^).

#### Soil water consumption

Surface runoff and drainage were assumed to be negligible because the experimental field was flat. The amount of ground water reaching the root zone was also negligible. Therefore, ET (mm) was calculated using the soil water balance Eq. ^33^:

3 $$ET = {\text{P}} + \Delta {\text{SW}}$$

where evapotranspiration (ET) and P are water consumption (mm) and effective precipitation (mm), respectively, during crop growth, and ∆SW (mm) is the change in soil water storage (mm).

#### Water use efficiency

The following equation was used to calculate WUE^34^:

4 $$WUE = Y/ET$$

where *WUE* is water use efficiency (kg ha^−1^ mm^−1^), *Y* is grain yield (kg ha^−1^), and *ET* is total consumption (mm) during the growing season.

#### Yield and yield components

At physiological maturity, each replicate plot was harvested manually to determine soybean grain yield. Pod number per plant, seed number per pod, and 100-grain weight were calculated from ten random samples per plot.

#### Net economic profit

Net economic profit for each treatment was calculated as follows^35^:

5 $$OV = Y \times P$$

6 $$IV = LC + MC + MCC + SFC$$

7 $$NI = OV - IV$$

where *OV* is output value [Chinese Yuan (CNY) ha^−1^], *Y* is grain yield (kg ha^−1^), *P* is local price of soybean grain (CNY ha^−1^), *IV* is total input value (CNY ha^−1^), *LC* is labor cost (CNY ha^−1^), *MC* is film mulch cost (CNY ha^−1^), *MCC* is machine-cultivation cost (CNY ha^−1^), *SFC* is seed and fertilizer cost (CNY ha^−1^), and *NI* is net income (CNY ha^−1^).

### Statistical analysis

Data (soil temperature, total ET, WUE, grain yield, yield components, and net economic profit) were statistically analyzed (ANOVA) using SPSS statistical software (v.17.0). Treatment mean values were compared using Fisher’s least significant difference (LSD) at P < 0.05.

#### Statement

“Longhuang No. 3”, the soybean (G*lycine max(Linn*.) Merr.) cultivar that we used in the present experiment, complied with international guidelines. We complied with the IUCN Policy Statement on Research Involving Species at risk of extinction and the Convention on the Trade in Endangered Species of Wild Fauna and Flora.

## Results

### Soil temperature

**Figure 2:**
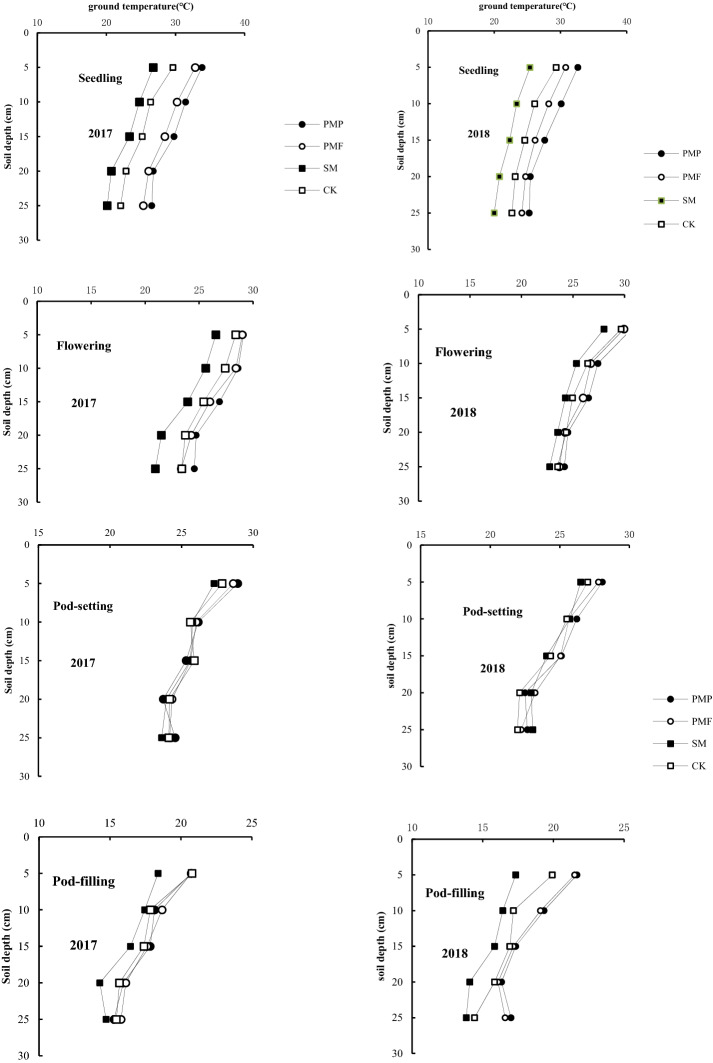

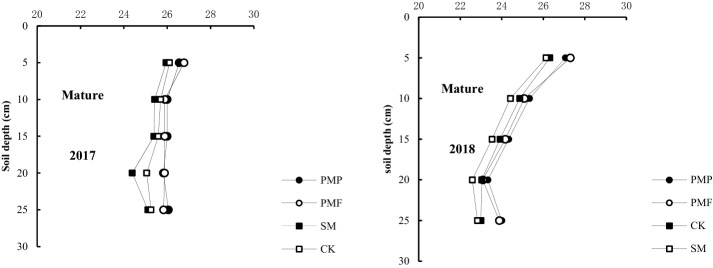
Soil temperature (°C) in the 0–25 cm layer during the 2017 and 2018 soybean growing seasons under different cultivation practices.

The top soil temperature (0–25 cm) differed between treatments, with the SM treatment significantly decreasing soil temperature and the PMF and PMP treatments increasing soil temperature (Fig. 2). Across the whole growing season, the SM treatment significantly reduced the average soil temperature by 1.15 °C in 2017 and 0.96 °C in 2018 compared with CK. In contrast, the PMP and PMF treatments significantly increased the average soil temperature by 1.30 and 1.31 °C in 2017 and 0.76 and 1.00 °C in 2018, respectively, compared with CK. In addition, the soil temperatures in the mulching treatments varied with growth stage. During early growth (seedling), soil temperatures increased by 3.03–4.47 °C and 1.63–3.38 °C in the PMP and PMF treatments and decreased by 2.07–2.80 °C in the SM treatment compared with CK. During mid-growth (flowering to podfilling), soil temperature increased by 0.22–1.48 °C and 0.23–1.23 °C in the PMP and PMF treatments and decreased by 0.28–1.96 °C in the SM treatment compared with CK. At maturity, the soil temperature decreased by 0.28–0.33 °C in the SM treatment and increased by 0.54–0.58 °C and 0.47–0.51 °C in the PMP and PMF treatments compared with CK.

### Soil water consumption

Soybean water consumption varied between years (Table 2). Total ET ranged from 415.6–439.8 mm in 2017 to 432.5–455.7 mm in 2018. The PMP and PMF treatments had higher total ET than the other treatments, increasing by 22.1 and 24.2 mm in 2017 and 16.8 and 23.2 mm in 2018 compared to CK. The SM treatment had 12.3 mm higher total ET than CK in 2017 but did not significantly differ in 2018. The plastic film mulching and SM treatments significantly differed for ET in 2017 but not in 2018.

**Table 2:** Soil water consumption and water use efficiency under different cultivation practices in Hesheng County of Gansu Province, China, in 2017 and 2018.

Year	Treatment	Soil water storage before sowing (mm)	Soil water storage after harvest(mm)	Precipitation during the growing season (mm)	Total ET (mm)	WUE (kg ha^−1^ mm^−1^)
2017	PMP	224.8	182.4c	395.3	437.7a	8.5a
	PMF	224.8	180.3c	395.3	439.8a	8.8a
	SM	224.8	192.2b	395.3	427.9b	7.8b
	CK	224.8	204.5a	395.3	415.6c	6.4c
2018	PMP	189.6	153.3ab	413.0	449.3a	8.3a
	PMF	189.6	146.9b	413.0	455.7a	8.5a
	SM	189.6	157.6ab	413.0	445ab	7.6b
	CK	189.6	170.1a	413.0	432.5b	6.5c

Different letters indicate significant differences (P < 0.05) between treatments for soil water storage after harvest, total ET, and WUE.

### Water use efficiency

The mulching treatments significantly improved WUE in both years. The average WUE in each treatment was ranked PMF > PMP > SM > CK (Table 2). In 2017, the PMF treatment had 37.5 % higher WUE than CK and 3.5 and 12.8 % higher WUE than the PMP and SM treatments, respectively. In 2018, the PMF, PMP, and SM treatments had 30.8, 27.7, and 16.9 % higher WUE than CK, respectively, but the three mulching treatments did not significantly differ. In both years, the SM treatment had 19.8 % higher WUE than CK and 12.3 and 9.1 % lower WUE than the PMF and PMP treatments (no significant difference between PMF and PMP).

The mulching practices improved soil moisture and changed soil water consumption in different soil layers compared with CK (Table 3). In both years, soil water consumption in the 0–120 cm soil layer was 12.3–12.5 mm in the SM treatment and 20.0–23.2 mm in the PMF and PMP treatments, significantly higher than CK. For all treatments, the consumption of stored soil moisture mainly occurred in the 60–120 cm soil layer; the PMP and PMF treatments consumed more soil water (2.2–12.5 mm and 12.7–17.0 mm, respectively) than CK, while the SM treatment consumed 2.6–3.0 mm more than CK. The percentage of consumed soil water to total soil water content in the 60–120 cm soil layer was ranked CK>PMP>PMF>SM in 2017 and CK>PMF>SM>PMP in 2018, with the water consumption in 2018 higher than in 2017.

**Table 3:** Consumed water storage in different soil layers under different cultivation practices in Hesheng County of Gansu Province, China, in 2017 and 2018.

Year	Treatment	Soil water consumption (mm) at different soil depth						Total consumed of soil water storage
		0–20	20–40	40–60	60–80	80–100	100–120	
2017	PMP	1.3a	3.5a	4.2ab	13.2a	12.3a	7.9b	42.4a
	PMC	2.8a	3.1a	5.0a	10.4a	13.5a	9.7a	44.5a
	SM	0.3ab	2.6ab	6.2a	9.5ab	6.7b	7.3b	32.6b
	CK	− 2.9b	− 1.2b	3.5b	7.7b	7.3b	5.9c	20.3c
2018	PMP	1.9a	6.9a	8.7a	9.2a	7.0b	2.6c	36.3a
	PMC	− 2.5b	4.4ab	7.2a	8.7a	14.6a	10.3a	42.7a
	SM	1.7a	4.9a	5.8b	7.0ab	9.3ab	3.3c	32.0ab
	CK	− 1.3b	− 2.7b	6.9ab	5.5b	5.3b	5.8b	19.5b

Different letters indicate significant differences (P < 0.05) between treatments for soil water consumption and total consumption of soil water storage.

### Grain yield and yield components

The mulching treatments significantly affected soybean yield (Table 4). Pod number per plant, 100-seed weight, basic seedlings per hectare, and grain yield varied between the four treatments (Table 4). Across both years, the PMP, PMF, and SM treatments had 35.7, 41.5, and 23.5 % higher average grain yield than CK (P < 0.05), respectively (PMP and PMF treatments did not significantly differ). Pod number per plant increased by 41.3 and 37.1 % in 2017 and 40.1 and 39.4 % in 2018 in the PMP and PMF treatments and 15.0 and 17.8 % in the SM treatment, respectively, compared with CK. The SM and CK treatments had 2.5–3.8 % and 2.5 % more basic seedlings per hectare than the PMP and PMF treatments (PMF and SM treatments did not significantly differ in 2018). The four treatments did not differ for 100-grain weight in 2017 and 2018.

**Table 4:** Grain yield and yield components under different cultivation practices in Hesheng Town of Gansu Province, China, in 2017 and 2018.

Year	Treatment	Pod number per plant	Seed number per pod	100-seed weight (g)	Basic seedlings per hectare (10^4^ ha^−1^)	Yield (kg ha^−1^)
2017	PMP	40.4a	2.0a	28.6a	15.8c	3705.2a
	PMF	39.2a	1.9a	28.4a	16.1b	3856.4a
	SM	32.9b	2.0a	28.3a	16.4a	3351.7b
	CK	28.6c	1.9a	28.0a	16.5a	2673.5c
2018	PMP	40.9a	2.1a	27.4a	16.2b	3716.5a
	PMF	40.7a	2.0a	28.5a	16.4ab	3878.4a
	SM	34.4b	2.0a	27.9a	16.5a	3403.8b
	CK	29.2c	2.0a	27.6a	16.6a	2794.7c

Different letters within columns indicate significant differences (P < 0.05) according to the LSD test.

### Economic returns

The price of Gansu soybean in 2017 and 2018 was 3900 and 3800 CNY t^−1^, respectively. Mulching increased the benefits of soybean planting but the methods differed due to differences in input costs and economic returns (Table 5). In both years, the PMP and PMF treatments increased MC, LC, and MMC inputs by 1155.0, 350.0, and 375.0 CNY ha^−1^, respectively, compared with the CK and SM treatments. The SM treatment had 501.3 and 2244.9 CNY ha^−1^ higher NI than the PMP and CK treatments in 2017, and 691.7, 76.5, and 1914.5 CNY ha^−1^ higher NI than the PMP, PMF, and CK treatments in 2018, respectively. On average, the SM treatment had 596.5 and 2079.7 CNY ha^−1^ higher NI than the PMP and CK treatments, but the PMF and SM treatments did not significantly differ.

**Table 5:** Economic returns and net income under different cultivation practices in Hesheng County of Gansu Province, China, in 2017 and 2018.

Year	Treatment	IV (CNY ha^−1^)					OV (CNY ha^−1^)	NI (CNY ha^−1^)
		MC	LC	MMC	SFC	Total		
2017	PMP	1155.0	3750.0	975.0	1815.0	7695.0	14,450.3ab	6755.3b
	PMF	1155.0	3750.0	975.0	1815.0	7695.0	15,040.0a	7345.0a
	SM	0.0	3400.0	600.0	1815.0	5815.0	13,071.6b	7256.6a
	CK	0.0	3000.0	600.0	1815.0	5415.0	10,426.7c	5011.7c
2018	PMP	1155.0	3750.0	975.0	1815.0	7695.0	14,122.7a	6427.7b
	PMF	1155.0	3750.0	975.0	1815.0	7695.0	14,737.9a	7042.9a
	SM	0.0	3400.0	600.0	1815.0	5815.0	12,934.4b	7119.4a
	CK	0.0	3000.0	600.0	1815.0	5415.0	10,619.9c	5204.9c

Different letters within columns indicate significant differences (P < 0.05) according to the LSD test.

## Discussion

### Soil temperature

The soil thermal regime affects soybean growth, development, and yield^35^. As a soil management measure, mulching can noticeably influence soil temperature^36,37^. However, the effects of mulching on soil temperature differ according to the mulching material used^38^. Plastic film mulching can significantly improve soil temperature at different crop growth stages^8,39^. Li et al. reported that ridge–furrow planting with plastic film mulch increased soil temperature by 2.6 °C in the ridges at the seedling stage, increasing soybean production by 44 %, compared to the conventional plat tillage without mulching^40^. Kader et al. reported that black plastic film mulch increased soil temperature and yield of soybean by 1–4 °C and 31–3 % compared to silver and transparent plastic mulch^41^. Luo et al. found that combined black plastic film and straw mulch, transparent plastic film and straw mulch, sole black plastic film mulch, and sole transparent plastic mulch significantly increased topsoil temperature in wheat production^42^. Straw mulch significantly reduced soil temperature in potato and maize production compared with the warming effect of plastic film mulch^43,44^. Our study showed that the film mulch treatments significantly increased soil temperature in the 0–25 cm layer by 0.76–1.31 °C during the growing season, consistent with Lu et al.^44^. During early growth from sowing to flowering, we found that the soil temperature increased from 1.63 to 4.47 °C, favorable for soybean growth. The PMP treatment had a warming effect on soil temperature, increasing by 1.09–1.40 °C at the seedling stage compared with the PMF treatment, resulting in seedling death and a significant reduction in the basic seedlings per hectare (down 1.9 %) at the cotyledon stage in 2017. Compared with the other treatments, the PMP treatment likely increased soil temperatures because the ridge–furrow with plastic film mulch had a larger plastic film area to receive solar radiation^45^. In contrast, the SM treatment significantly reduced the average soil temperature by 0.96–1.15 °C in the 0–25 cm layer compared with CK, similar to Chang et al., who reported that maize straw mulch had the lowest temperature during some growth stages in potato^46^. The straw mulching likely formed an isolation layer between solar and earth thermal radiation, preventing heat exchange between them, compared with traditional bare land planting without mulching, the straw mulching treatment decreased soil temperature by 0.8–1.4 °C, and increased potato yield by 10.5–34.2 % (Chang et al.)^46^.

### Soil water

Mulching improves soil water retention and inhibits soil evaporation in dry and semi-arid areas. Chang et al. reported that bundled straw mulching improved moisture by significantly increasing water consumption during the soybean growing season^46^. Zhang et al. found that mulching increased the consumption ratio of deep water from below the 120 cm soil layer; plastic film mulch and straw mulch significantly increased WUE in winter wheat by 9.8–13.9 %, and 18.4–22.0 %, respectively^47^. Ren et al. reported that plastic-covered ridges in a ridge–furrow farming system significantly increased soil moisture storage in the top 0–100 cm layer during the corn-growing season^48^. Akhtar et al. concluded that straw mulching (6 Mg ha^−1^) changed the soil hydrothermal regime, favoring soybean growth when supplied with 27 kg N ha^−1^in semi-arid northwest China^49^. In our study, total ET ranged from 415.6 to 455.7 mm during the soybean growing season, and the PMP and PMF treatments had significantly higher total ET than the SM and CK treatments. Our studies showed that soil water consumption mainly occurred in the 60–120 cm soil layer, with the PMP and PMF treatments consuming much more soil water than the CK and SM treatments, possibly because mulching increases root length and root activity^50^. The SM treatment had 19.8 % higher WUE than CK, which was 12.3 and 9.1 % lower than the PMF and PMP treatments, consistent with the findings of Kader et al. on soybean^51^, Zhang et al. on spring maize^52^, Li et al. on winter wheat^40^, and Chang et al. on potato^46^.

### Grain yield

Mulching is effective for resisting drought stress in agricultural production in semi-arid areas. Plastic film mulch can decrease soil water evaporation and increase soil temperature and WUE^52^, increasing grain yield and economic benefit^10,41^. Straw mulching affects soil enzyme activity and soil properties, improving the soil micro-environment and thus yield^49^. Studies have found that straw mulching increases soil moisture retention^53^, decreases soil salinity and increases soil organic matter^54^, and improves the root growth environment and crop yield and productivity^55^. The plastic and straw mulching can improve emergence in soybean in both normal and crusted soils, possibly by conserving soil moisture^56^. In our study, mulching significantly increased soybean grain yield (PMF>PMP>SM>CK). The water conservation ability of mulch at the flowering stage likely promoted flowering and pod formation^53^. The PMP, PMF, and SM treatments increased soybean grain yield by 35.7, 41.5, and 23.5 %, respectively, compared with CK. The grain yield under plastic film mulch and straw mulch significantly differed by 12.8 % in 2017 and 11.6 % in 2018, with the yield under plastic film mulch higher than under straw mulch. Kader et al. reported 31–34 % higher soybean yields under colored film mulch than bare soil^41^.

### Economic returns

Efficiency and returns are the most important goals of agricultural production. The planting benefit analysis of the four treatments in our study revealed that plastic film mulch had the highest net income (average 14,587.7 CNY ha^−1^), being 2479.7 CNY ha^−1^ (12.2 %; not significant) and 4365.7 CNY ha^−1^ (38.6 %) higher than straw mulch and bare soil, respectively. These findings agree with Zhang et al., who reported that film-mulched ridges, furrow-flat planting, and alternating film-mulched ridges increased net income in maize^34^. However, in China utilization of crop straw is very low^57^. Straw mulch is also cheaper to implement, further increasing the economic benefits of the production systems^58^.

## Conclusions

Straw mulching improved soil moisture, soil water content, and WUE and maintained the economic returns of soybean cultivation compared to plastic film mulching. Strip straw mulching could be used as an environmentally friendly cultivation technology for soybean production in semi-arid regions of the Loess Plateau in China.

## Supplementary Information

Supplementary Information.

## Data Availability

All data generated or analyzed during this study are included in this published article and its Supplementary Information files.
